# Chronic *Toxoplasma gondii* Infection Exacerbates Secondary Polymicrobial Sepsis

**DOI:** 10.3389/fcimb.2017.00116

**Published:** 2017-04-07

**Authors:** Maria C. Souza, Denise M. Fonseca, Alexandre Kanashiro, Luciana Benevides, Tiago S. Medina, Murilo S. Dias, Warrison A. Andrade, Giuliano Bonfá, Marcondes A. B. Silva, Aline Gozzi, Marcos C. Borges, Ricardo T. Gazzinelli, José C. Alves-Filho, Fernando Q. Cunha, João S. Silva

**Affiliations:** ^1^Department of Biochemistry and Immunology, Ribeirão Preto Medical School, University of São PauloSão Paulo, Brazil; ^2^Department of Pharmacology, Ribeirão Preto Medical School, University of São PauloSão Paulo, Brazil; ^3^Department of Medicine, University of Massachusetts Medical SchoolWorcester, MA, USA; ^4^Department of Internal Medicine, Ribeirão Preto Medical School, University of São PauloSão Paulo, Brazil

**Keywords:** sepsis, septic shock, *Toxoplasma gondii*, coinfection, chronic disease

## Abstract

Sepsis is a severe syndrome that arises when the host response to an insult is exacerbated, leading to organ failure and frequently to death. How a chronic infection that causes a prolonged Th1 expansion affects the course of sepsis is unknown. In this study, we showed that mice chronically infected with *Toxoplasma gondii* were more susceptible to sepsis induced by cecal ligation and puncture (CLP). Although *T. gondii*-infected mice exhibited efficient control of the bacterial burden, they showed increased mortality compared to the control groups. Mechanistically, chronic *T. gondii* infection induces the suppression of Th2 lymphocytes via *Gata3*-repressive methylation and simultaneously induces long-lived IFN-γ-producing CD4^+^ T lymphocytes, which promotes systemic inflammation that is harmful during CLP. Chronic *T. gondii* infection intensifies local and systemic Th1 cytokines as well as nitric oxide production, which reduces systolic and diastolic arterial blood pressures after sepsis induction, thus predisposing the host to septic shock. Blockade of IFN-γ prevented arterial hypotension and prolonged the host lifespan by reducing the cytokine storm. Interestingly, these data mirrored our observation in septic patients, in which sepsis severity was positively correlated to increased levels of IFN-γ in patients who were serologically positive for *T. gondii*. Collectively, these data demonstrated that chronic infection with *T. gondii* is a critical factor for sepsis severity that needs to be considered when designing strategies to prevent and control the outcome of this devastating disease.

## Introduction

The majority of studies regarding host-pathogen relationships have focused on the interaction of a single pathogen with its host. However, humans are commonly exposed recurrently or simultaneously to multiple pathogens (Jamieson et al., 2010; Telfer et al., 2010). Understanding how previous/simultaneous infections can modify the host immune response and consequently affect the outcome of a secondary infection is crucial to designing new therapeutic strategies to control coinfections.

Sepsis, a life-threatening disease associated with high morbidity and mortality worldwide, is caused by a dysregulation of the immune system to different infectious agents (Schmid et al., 2004). An uncontrolled infection induces a systemic inflammatory reaction that culminates in a cytokine storm. Such inflammatory mediators deregulate the cardiovascular system, cause vascular permeability, and lead to severe sepsis and septic shock characterized by severe hypotension, multi-organ failure, and death (Bone et al., 1997; Hotchkiss et al., 2009).

Toxoplasmosis is a highly prevalent protozoan infection, affecting approximately one-third of the world population (Robert-Gangneux and Darde, 2012). The oral route of infection favors the disruption of the intestinal epithelium caused by inflammatory stressors induced against the parasite and facilitates the spread of the parasite toward different target organs (Dubey et al., 1998; Montoya and Liesenfeld, 2004). Locally, innate cells express sensors such as Toll-like receptors (TLRs) that are indispensable to parasite recognition (Debierre-Grockiego et al., 2007; Andrade et al., 2013). *T. gondii* infection induces a scenario of intense inflammation, characterized by IFN-γ-producing long-lived CD4^+^ and CD8^+^ T lymphocytes (Gazzinelli et al., 1994; Mashayekhi et al., 2011; Hand et al., 2012). Although the parasite growth is controlled efficiently, it triggers intense tissue damage that is commonly harmful to the host (Mashayekhi et al., 2011). After the acute phase of infection, the parasite is maintained latently in the brain and skeletal muscle, leading to a chronic state (Munoz et al., 2011). This evidence suggests that long-lived *T. gondii*-induced lymphocytes have an intrinsic molecular programme that is promptly activated to release excessive amounts of IFN-γ after a secondary pathogenic exposure. Although clear evidence exists that sepsis modulates secondary infections (Nascimento et al., 2010), it is currently unknown whether previous infections can interfere with the sepsis outcome.

In this study, we showed that IFN-γ-producing lymphocytes induced by *T. gondii* parasites persist after the acute phase of infection and are deleterious during polymicrobial sepsis. Mechanistically, *T. gondii* infection is followed by *Gata3* methylation and increased transcription of IFN-γ-related genes in CD4^+^ T cells, thus inducing long-lived memory T cells. The partial blockage of IFN-γ prevented massive cytokine production, arterial hypotension, and prolonged host lifespan. Notably, these data mirrored our observation in patients because elevated serum levels of IFN-γ correlate with sepsis severity. Additionally, patients serologically positive for *T. gondii* had increased serum levels of IFN-γ compared to patients who were serologically negative. These observations demonstrate that chronic infection with *T. gondii* aggravates the course of sepsis and opens new avenues to design strategies to control the severity of *T. gondii*-sepsis coinfection.

## Materials and methods

### Ethics statement

The research was approved by the Ethics Committee on Animal Experiments of Ribeirão Preto Medical School (CETEA-107-2009) and the Institutional Animal Care and Use Committee of the University of Massachusetts Medical School (IACUC-2371-12). The study on human samples was approved by the Human Research Ethics Committee at Hospital das Clínicas of Faculdade de Medicina da Universidade de São Paulo (research protocol n°. 536/2008). After obtaining written informed consent from all of the patient's relatives, venous blood samples were collected.

### Mice and parasites

Female C57BL/6 mice were used. The low-virulent ME-49 strain of *T. gondii* was harvested from the brains of infected mice (Benevides et al., 2008). Since our major aim was to address sepsis susceptibility in chronically infected mice, we only used the ME-49 strain that allows mice to progress to chronic phase of infection.

### Polymicrobial sepsis model

Sepsis was induced using a cecal ligation and puncture (CLP) model. Two punctures with sterile 21-G needles were used to standardize the sub-lethal sepsis (Benjamim et al., 2003; Nascimento et al., 2010).

### Bacteria count determination and leukocyte migration to the peritoneum

The quantification of the bacterial load in the blood and peritoneal exudates was performed at 6, 12, and 24 h after CLP. For these analyses, the animals were anesthetized, and blood was collected via cardiac puncture, following which the animals were euthanized in a CO_2_ chamber. The peritoneal exudates were collected via an injection of 1.5 mL of PBS/EDTA into the peritoneal cavity. After the sample collection, 10 μL of blood or peritoneal wash without dilutions were plated on Mueller-Hinton agar (Difco Laboratories, Detroit, MI, US) and incubated at 37°C under aerobic conditions for 24 h. Colony-forming units (CFUs) were expressed as Log2 of CFU/10 μL of blood or peritoneal wash. All procedures were performed under sterile conditions (Nascimento et al., 2010). Leukocyte migration and differential count were assessed 6, 12, and 24 h after the induction of CLP. The cells present in the peritoneal cavity were harvested via washing of the peritoneal cavity using 1.5 ml of phosphate buffered saline (PBS) containing EDTA (1 mM). The total leukocyte counts were obtained with a cell counter (Coulter AC T series analyser, Coulter, Miami, FL), and the differential count was performed using flow cytometry (BD Immunocytometry System, Franklin Lakes, NJ, USA). The results were expressed as the number of total leukocytes, neutrophils or lymphocytes in the peritoneal cavity (Alves-Filho et al., 2006).

### Histopathological analyses

For pathological analyses, the gut was washed to remove the intestinal contents, and the ileum fragment was individually wrapped in a “Swiss roll,” fixed in 10% formalin, embedded in paraffin and processed routinely for haematoxylin and eosin staining. Slides were imaged using light microscopy. The images were acquired with a digital camera (Leica DC300F, Switzerland) coupled to a microscope for histological analysis.

### Flow cytometry

All antibodies used for flow cytometry were purchased from BD Biosciences or eBiosciences and used according to the manufacturer's instructions. For neutrophil staining, Ly6G (FITC, 1A8), CD11c (APC-Cy7, N418), CD11b (PE Cy7, M170) and F4/80 (Percp Cy5, BM8) were used. For intracellular cytokine staining, CD3 (FITC, 145-2C11), CD4 (PercP, RM4-5), CD8 (PE Cy7, 53-6.7), IFN- γ (APC, XMG1.2) and TNF-α (PE, MP6X722) were used. For transcription factors, CD3 (FITC, 145-2C11), CD4 (APC Cy7, RM4-5), CD44 (PercP, IM7) and Tbet (Alexa 647, 4B10) or CD3 (PE, 145-2C11), CD4 (APC Cy7, RM4-5), CD44 (PercP, IM7), RorγT (APC, Q31-378) and Gata3 (Alexa 488, L50-823) were used. For memory cells, CD3 (PE, 145-2C11), CD4 (APC Cy7, RM4-5), CCR7 (Alexa 647, 3D12) and CD62L (FITC, MEL-14) were used. Briefly, tissue-isolated cells were incubated with monoclonal antibodies in buffer containing blocking antibody. For intracellular staining, the cells were harvested and plated with PMA-ionomycin and Brefeldin A (Golgi plug BD bioscience), and stained for flow cytometry analysis. After incubation, cells were fixed and permeabilized with the BD Cytofix/Cytoperm kit (BD Biosciences and eBiosciences, CA, USA). Cell acquisition was performed on the BD CANTO II cell analyser (BD Biosciences) using FACSDiva software (BD Biosciences), and data were analyzed using FlowJo software (Tree Star, Ashland, OR).

### Measurement of cytokine and chemokine levels

The levels of mouse TNF-α, IFN-γ, IL-6, and KC were measured using DuoSet ELISA kits (R&D Systems).

### Induction of colitis

Chronic dextran sodium sulfate (DSS) colitis was induced by administering 3% (w/v−1) DSS (molecular mass, 36–50 kDa; MP Biomedicals, OH) for 14 days, followed by 26 days of normal drinking water.

### Antibody treatment

The cytokine IFN-γ was neutralized by intraperitoneal (i.p.) injection of 10 μg of purified mAb 24 h before CLP. The mouse anti-IFN-γ mAb was purified from the ascites of mice injected with an anti-IFN-γ hybridoma (XMG1.2). Controls received 500 μg of normal rat IgG diluted in PBS. The IgG was isolated from naïve rats using protein G column purification.

### Antibiotic treatment

The broad-spectrum antibiotic treatment regimen was followed as described by Hand et al. (2012) with modifications. Briefly, each animal received a daily combination of 5 mg neomycin trisulfate (Sigma), 2.5 mg vancomycin (Sigma), 5 mg metronidazole (sigma) and 5 mg ampicillin sodium (sigma) diluted in 200 μL water via oral gavage for 2 weeks before and 2 weeks post-infection with *T. gondii*.

### Epigenetics analysis

For cell isolation, CD4^+^ T cells were negatively selected using the EasySep kit (StemCell Technologies, Canada), and DNA extraction was subsequently performed. The epigenetic analysis was performed using the PCR kit EpiTect Methyl II PCR Arrays (Qiagen Sciences, USA) to determine the methylated CpG islands and indicate the percentage of methylated DNA. The DNA in each individual enzymatic reaction was quantified using real-time PCR with primers that flanked the promoter region of interest as follows: *tbx21*- Chr11, CpG-start 96975880, CpG-end 96976654; *eomes*- Chr9, CpG-start 1183859814, CpG-end 118386331; *gata3*- Chr2, CpG-start 9802719, CpG-end 9803061 and *rora*- Chr9, CpG-start 68501352, CpG-end 68502769.

### Blood pressure analysis

Arterial blood pressure was non-invasively measured by determining the tail blood volume with a pressure recording sensor and an occlusion tail cuff (CODA System, Kent Scientific, CT) 24 h after CLP. The results were expressed in millimeters of mercury (mmHg).

### Patient samples and experiments

The patients' blood was collected in the first 24 h after admission. The samples were processed and analyzed for cytokine production and *Toxoplasma gondii* serology. The levels of the cytokine IFN-γ were measured using DuoSet ELISA as indicated by the manufacturer's instructions (R&D Systems). The *Toxoplasma gondii* serology was performed by indirect immunofluorescence assay as indicated by the manufacturer's instructions (FLUOCON IgG/IgM WAMA, Belgium).

### Statistics

The animal survival was expressed as the percentage of surviving animals analyzed using the Mantel-Cox log-rank test (X_2_, chi-squared). For comparison of multiple parametric data, the variance (ANOVA) tests were used followed by the *post-hoc* Tukey-Kramer test. Data are expressed as the means ± standard error of the mean (SEM). Statistical analysis and graphics were performed using the GraphPad Prism version 5.0 (GraphPad Software, San Diego, CA, USA).

## Results

### Chronic *T. gondii* infection increases the susceptibility to polymicrobial sepsis

**Figure 1:**
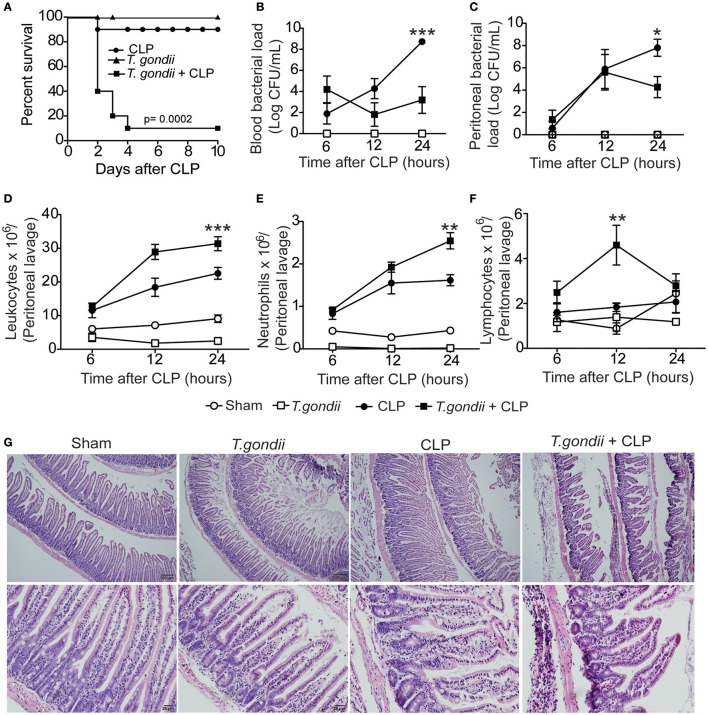
**Chronic *T. gondii* infection aggravates polymicrobial sepsis**. Forty days after *T. gondii* infection, C57BL/6 mice were subjected to CLP for different analyses. The survival rate was evaluated until the 10th day post-CLP induction **(A)**. These data are representative of three independent experiments (*n* = 10), and the statistical analysis was determined using the Mantel-Cox log-rank test. The bacterial load was analyzed in the blood **(B)** and peritoneal lavage **(C)** at 6, 12, or 24 h post-CLP induction. The results are expressed as the log of the colony-forming units (CFU) per microliter. The leukocytes from the peritoneum **(D)** were stained with May-Grünwald Giemsa, and the number of neutrophils **(E)** and lymphocytes **(F)** were determined using a cell counter. Data are presented as the means ± SEM for 4 animals per group in four independent experiments (ANOVA, followed by Tukey's test; ^*^p < 0.05; ^**^p < 0.01; ^***^p < 0.001). At 24 h post-CLP, histopathological features of the ileum were analyzed after staining with haematoxylin and eosin (H&E). The results are representative of three independent experiments using 4 mice per group **(G)**.

Mice chronically infected with *T. gondii* for 40 days were subjected to sublethal sepsis induced by CLP surgery (coinfected mice). Herein, we observed increased mortality of coinfected mice compared to sublethal CLP-subjected mice (Figure 1A). These data indicate that chronic *T. gondii* infection aggravated polymicrobial sepsis, which was not due to toxoplasmosis reactivation (Figures S1A,B).

To investigate whether coinfected mice were able to control bacterial proliferation, the bacterial burden was quantified in the blood and peritoneum. We found that 24 h after CLP, coinfected mice were more efficient in controlling bacterial replication both systemically (Figure 1B) and locally (Figure 1C) compared to septic mice. Moreover, we counted the number of leukocytes recovered from the peritoneal cavity after CLP. The total leukocyte count strikingly decreased in the blood of coinfected mice (data not shown); however, the leukocytes increased in the peritoneum of coinfected mice compared to septic mice (Figure 1D). The neutrophil recruitment to the peritoneal cavity was increased in coinfected mice compared to CLP-subjected mice (Figure 1E), which paralleled the bacterial clearance. We also observed increased migration of lymphocytes in mice 12 h after CLP (Figure 1F). To investigate whether intense cellular recruitment to the peritoneal cavity reflects intestinal inflammation and tissue damage, we evaluated the histological features in the intestine 24 h after sepsis induction. We found extensive neutrophil infiltrate mainly in the ileum of coinfected mice along with decreased mRNA expression of occludin, a tight junction protein (Figures S2A,B), and massive intestinal tissue damage (Figure 1G). These data suggest that chronic *T. gondii* infection promotes a microenvironment in the intestine that favors the inflammatory response during CLP. Notably, we did not find evidence of vital organ failure in coinfected mice, which was evaluated by either histopathological analysis or biochemical biomarkers for renal, hepatic, cardiac, and muscular dysfunctions.

### Chronic *T. gondii* infection intensifies proinflammatory cytokine production during sepsis

**Figure 2:**
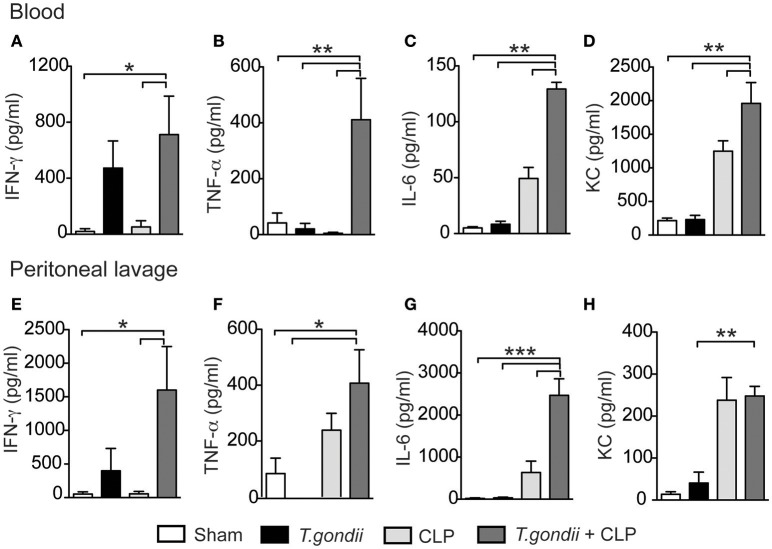
**The *T. gondii*-primed immune response promotes a storm of cytokines associated with CLP**. C57BL/6 mice were infected with 5 cysts of *T. gondii*, and 40 days after infection, the mice were subjected to CLP. Twenty-four hours after CLP, the serum and peritoneal lavage were collected for cytokine analysis. The levels of pro-inflammatory cytokines IFN-γ **(A,E)**, TNF-ɑ **(B,F)**, IL-6 **(C,G)**, and the chemokine KC **(D,H)** were determined using ELISA. Data are presented as the means ± SEM for 4 animals per group in three different experiments (ANOVA followed by Tukey's test; ^*^p < 0.05; ^**^p < 0.01; ^***^p < 0.001).

**Figure 3:**
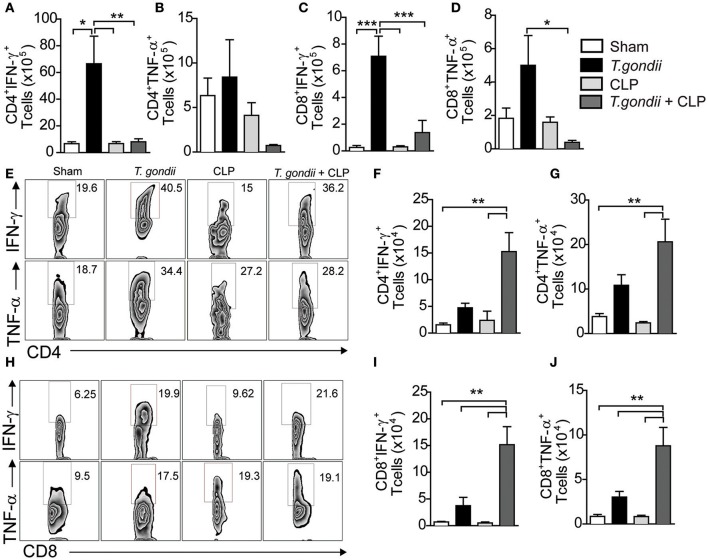
**The *T. gondii*-experienced CD4+ and CD8+ T cells are programmed to produce inflammatory mediators during sepsis**. Naïve or chronically *T. gondii*-infected mice were subjected to CLP to evaluate the frequencies of IFN-γ- or TNF-α-producing CD4^+^ and CD8^+^ T cells in the spleen **(A–D)**. Peritoneal leukocytes were recovered from the peritoneal lavage 24 h after CLP, then stimulated with PMA-ionomycin, and stained for flow cytometry analysis **(E–J)**. Data are presented as the means ± SEM for 4 animals in three different experiments. Statistical analysis was performed using ANOVA followed by Tukey's test; ^*^p < 0.05; ^**^p < 0.01; ^***^p < 0.001.

The major factor leading to host susceptibility during sepsis is the increased production of inflammatory cytokines, which can promote septic shock (Ebong et al., 1999). To evaluate whether the high mortality of coinfected mice mirrored the production of inflammatory mediators, we quantified the cytokines in the serum and peritoneal lavage 24 h after CLP. Notably, coinfected mice showed increased levels of IFN-γ (Figures 2A,E), TNF-α (Figures 2B,F), IL-6 (Figures 2C,G), and KC (Figures 2D,H) in both the serum and peritoneal lavage compared to the control groups. For the immuneregulatory cytokines, IL-4 was not detected and the levels of IL-10 in the peritoneal lavage were similar in all groups studied. Splenic CD4^+^ and CD8^+^ T cells of *T. gondii*-infected mice produced elevated levels of IFN-γ and TNF-α even in the absence of a secondary stimulus (Figures 3A–D). Although the size of the spleen from *T. gondii*-infected mice was similar to naïve mice, after the induction of sepsis, *T. gondii*-infected mice experienced a notable reduction in spleen size along with a striking emergence of IFN-γ- and TNF-α-producing CD4^+^ (Figures 3E–G) and CD8^+^ (Figures 3H–J) T cells into the peritoneum. These data suggest that inflammatory cells were leaving the spleen/bloodstream and reaching the peritoneal cavity. Thus, *T. gondii*-programmed CD4^+^ and CD8^+^ T cells may be recruited to the site of sepsis and are the primary source of inflammatory mediators during sepsis in infected mice.

### Long-lived CD4^+^ *T. gondii*-primed T cells release IFN-γ and TNF-α during sepsis

**Figure 4:**
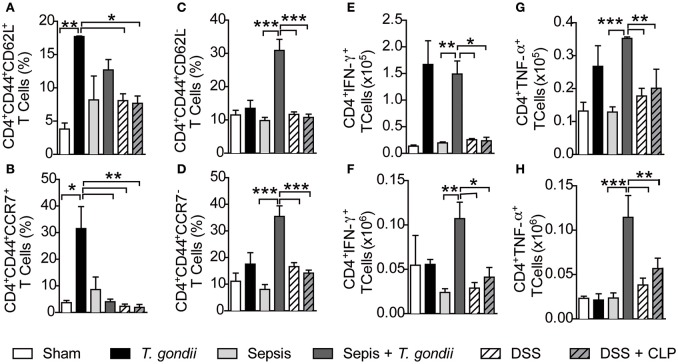
***T. gondii*****-infected mice induce long-lived CD4^+^ T cells that are reactivated during sepsis**. C57BL/6 mice were infected with 5 cysts of *T. gondii* or treated with 3% dextran sodium sulfate (DSS) to induce colitis. After 40 days of *T. gondii* infection or DSS treatment, the animals were subjected to CLP. The frequency of central memory-like (CD4^+^CD44^+^CD62L^+^ or CCR7^+^) **(A,B)** and effector memory-like (CD4^+^CD44^+^CD62L^−^ or CCR7^−^) **(C,D)** T cells was quantified in the mesenteric lymph nodes of control, coinfected, and colitis-induced mice. IFN-γ- or TNF-α-producing CD4^+^ T cells were recovered from the mesenteric lymph nodes **(E,G)** or peritoneal lavage and quantified **(F,H)**. Data are presented as the means ± SEM for 4 animals in three different experiments. The lymphocytes were analyzed using flow cytometry, and statistical analysis was performed using ANOVA followed by Tukey's test; ^*^p < 0.05; ^**^p < 0.01; ^***^p < 0.001.

To further gain insights into the mechanisms by which chronic *T. gondii* infection aggravates sepsis, we assessed whether chronic infection maintained a pool of long-lived lymphocytes that act as first responders in polymicrobial sepsis. As expected, chronic *T. gondii* infection maintained a pool of CD4^+^ and CD8^+^ T cells (CD4^+^CD44^+^ and CD8^+^CD44^+^ T cells, respectively) independent of sepsis (Figures S3A,B), thus confirming that such cells were activated by *T. gondii*. Indeed, chronic infection deeply induced the transcription of IFN-γ-related genes, which were intensified during sepsis development (Figure S3C). As long-lived T cells comprise a pool of central and effector memory cells (Wherry et al., 2003), we explored whether *T. gondii* exposure maintained a pool of memory T cells during its chronic phase. Chronic *T. gondii* infection induced an increased number of CD4^+^CD44^high^CD62L^+^CCR7^+^ T lymphocytes, named here as central memory-like T lymphocytes (Figures 4A,B) that were converted to effector memory-like (CD4^+^CD44^high^CD62L^−^CCR7^−^) T lymphocytes after CLP surgery (Figures 4C,D). Herein, sepsis induction reactivated long-lived *T. gondii*-experienced T cells to produce IFN-γ and TNF-α in both the mesenteric lymph nodes (Figures 4E,G) and peritoneum (Figures 4F,H). To evaluate the involvement of microbiota in promoting long-lived T cells, we induced colitis with a classical intestinal stressor dextran sodium sulfate (DSS) to promote bacterial translocation followed by CLP. Surprisingly, DSS-induced bacterial translocation was unable to induce central and effector memory-like T lymphocytes (Figures 4A–D) and IFN-γ and TNF-ɑ production after sepsis induction (Figures 4E–H). These results rule out a potential role of the microbiota in generating deleterious long-lived T cells that cross-react against released bacteria via CLP.

### Chronically *T. gondii*-infected mice develop hypotension during sepsis

**Figure 5:**
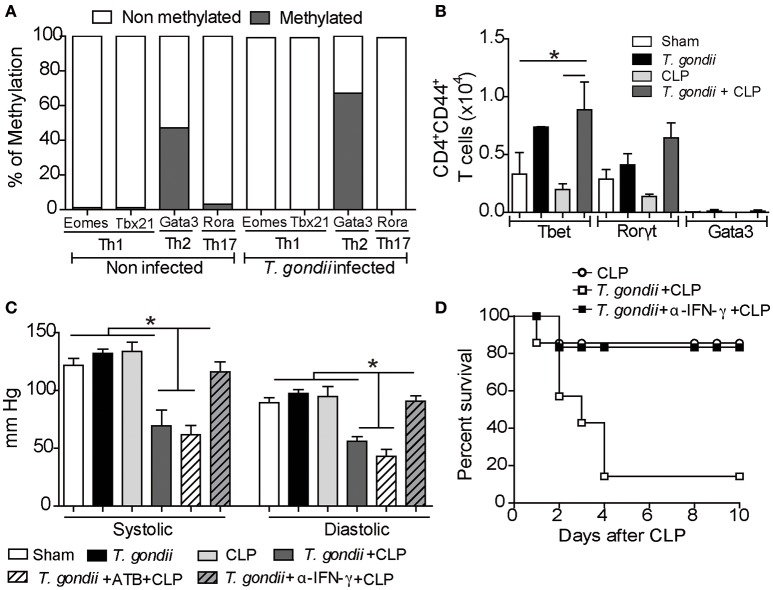
**Treatment with anti-IFN-γ prevents hypotension and ameliorates host survival**. For the methylation analysis, CD4^+^ T cells were separated from spleen cells by negative selection for DNA extraction. The pattern of DNA methylation was determined using the EpiTect Methyl II PCR Arrays kit. This analysis was performed in two independent trials, and the variation rate ≥ 10% was considered significant **(A)**. The expression of the transcription factors *T-bet, Gata-3* and *Ror*γ*T* was determined in CD44^high^CD4^+^ T cells using flow cytometry **(B)**. For blood pressure analysis, a group of coinfected mice was pretreated with broad-spectrum antibiotics (ATB) 2 weeks before and after *T. gondii* infection, and another group was treated with anti-IFN-γ antibodies 24 h before CLP induction. After 40 days of *T. gondii* infection, the mice were subjected to CLP, and at 24 h post-CLP, the blood pressure (mmHg) was evaluated in the animal tail using a sphygmomanometer **(C)**. The bars represent the means ± SEM for 4 animals per group (^*^p < 0.05). The survival assay was monitored for 10 days after treatment with a 10 μg/kg dose of anti-IFN-γ antibodies **(D)**. The results are expressed as a percentage of survival, and the *p*-value was considered when comparing chronic *T. gondii* infected/CLP mice who received antibody treatment vs. animals that were not treated.

*T. gondii*-primed CD4^+^ T lymphocytes are polarized toward a Th1 profile (Gazzinelli et al., 1994), and recently, it was demonstrated that IFN-γ suppresses permissive chromatin remodeling in the regulatory region of the *Il4* gene (Nishida et al., 2013). To test whether the microenvironment elicited by chronic toxoplasmosis could promote epigenetic reprogramming of lymphocytes, we purified splenic CD4^+^ T cells and investigated the repressive methylation status of their transcription factors. During the chronic phase of *T. gondii* infection, *Gata3* was found significantly methylated compared to the naïve CD4^+^ T splenic cells (Figure 5A). Additionally, repressive methylation of Th17- (*Rora*) and Th1-associated transcription factors (*Tbx21* and *Eomes*) were undetectable in chronically infected mice (Figure 5A). The epigenetic changes observed indicate that Th1-induced expression can be due to *Gata3* repression. This provides further evidence for our hypothesis that *Gata3* repression during *T. gondii* infection drives the immune response toward a Th1 pattern. To characterize whether CD4^+^ T lymphocytes are driven to long-lived Th1 lymphocytes during the chronic infection, we analyzed the transcription factors expressed by the Th1 lymphocytes (*Tbet*). We observed that the *T. gondii*-induced inflammatory milieu reprogrammed naïve CD4^+^ T lymphocytes into long-lived Th1 lymphocytes independent of CLP (Figure 5B). In contrast, *T. gondii*-primed T cells did not express significant amounts of the transcription factors *Ror*γ*t* and *Gata3* with or without a secondary infection, which excludes the role of long-lived Th17 and Th2 cells in our model.

Nitric oxide (NO) production and the subsequent cytokine storm induced during sepsis are major inductors of hypotension and septic shock (Bone et al., 1997). Indeed, we detected increased NO production in coinfected mice compared to CLP-subjected mice (Figure S4). Since coinfected mice also had increased inflammatory cytokines, mainly IFN-γ, we explored whether chronic *T. gondii* infection could aggravate sepsis by reducing systolic and diastolic arterial blood pressures. Only coinfected mice developed hypotension through the reduction of systolic and diastolic blood pressures (Figure 5C). Moreover, the partial blockage of IFN-γ prevented hypotension in these mice (Figure 5C).

Recent data have revealed that *T. gondii* infection promotes long-lived IFN-γ-producing microbiota-specific CD4^+^ T cells (Hand et al., 2012). To verify a possible role of the microbiota in controlling arterial blood pressure of coinfected mice, we treated them with broad-spectrum antibiotics to prevent bacterial translocation during the acute phase of *T. gondii* infection. We found that depletion of the microbiota did not prevent cytokine production and arterial hypotension (Figure 5C). These observations reveal that chronic *T. gondii* infection intensifies the plethora of cytokines during sepsis and predisposes the host to septic shock. Since the blockade of IFN-γ prevented host hypotension, we evaluated whether treatment with monoclonal anti-IFN-γ antibody could prevent the susceptibility of coinfected mice. The pretreatment with 10 μg/kg of anti-IFN-γ significantly improved the survival of these mice (Figure 5D).

### Positive serology for toxoplasmosis increases IFN-γ during sepsis

**Figure 6:**
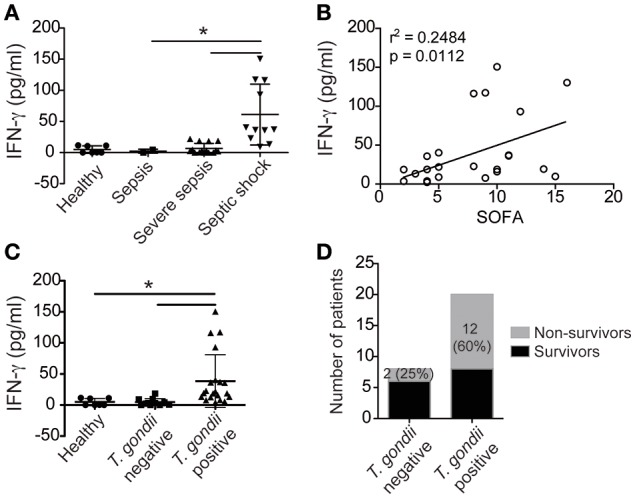
**Patients exposed to *T. gondii* have increased INF-γ during sepsis**. The serum concentrations of IFN-γ in septic patients or healthy individuals (healthy = 7, sepsis = 2, severe sepsis = 15, and septic shock = 11) were determined using ELISA **(A–C)**. The data shown are the mean values of individual subjects from triplicate experiments **(A–C)**. The statistical analysis was performed using ANOVA followed by Tukey's test; ^*^p < 0.05. Linear regression analysis of the means of IFN-γ from septic patients in relationship to changes in the Sepsis-related Organ Failure Assessment (SOFA) scores are shown (*r*^2^ = 0.2484; *p* < 0.0112) **(B)**. The number of non-survivors is presented as a percentage **(D)**.

To determine whether the IFN-γ-mediated mechanism described in coinfected mice is also observed in coinfected humans, we collected the blood of patients with sepsis to test the serology for toxoplasmosis and to measure IFN-γ production. Our data showed that patients with more severe clinical forms of sepsis had increased IFN-γ serum levels (Figures 6A,B), which strongly supports that IFN-γ production contributes to sepsis severity. In addition, patients serologically positive for toxoplasmosis had increased levels of IFN-γ during sepsis compared to serologically negative patients or healthy controls (Figure 6C). Similarly, previous exposure to *T. gondii* was deleterious to the host because mortality was increased in coinfected patients compared to patients who were serologically negative for toxoplasmosis (Figure 6D). Collectively, our data supports the hypothesis that patients with positive serology for toxoplasmosis are at risk for the development of severe sepsis.

## Discussion

Several studies demonstrated that a variety of outcomes is possible during a secondary infection, depending on the route of infection, the type of pathogen, or even the temporal proximity (Gardner, 1981; Navarini et al., 2006; Barton et al., 2007; Gumenscheimer et al., 2007; Humphreys et al., 2008; Miller et al., 2009; Jamieson et al., 2010; Fenoy et al., 2012). In this context, the involvement of a chronic infection followed by acute infections has been poorly explored. *T. gondii* creates a scenario of intense intestinal inflammation featured by the high prevalence of Th1 lymphocytes. Similarly, sepsis is a multifactorial disease characterized by systemic inflammatory response syndrome (SIRS). In the present study, considering their worldwide prevalence and their inflammatory condition, we conjectured that patients serologically positive for toxoplasmosis could aggravate the outcome of sepsis by intensifying the inflammatory response. Surprisingly, the majority of patients who died of septic shock were serologically positive for toxoplasmosis, which led us to investigate the cellular and humoral mechanisms involved in the immune response in mice that were chronically infected with *T. gondii* during sepsis development.

Leukocytes are required to control bacterial replication during a microorganism's invasion (Reddy and Standiford, 2010; Kovach and Standiford, 2012). Interestingly, although mice chronically infected with *T. gondii* were more susceptible to sepsis, they had better control of bacterial proliferation by enhancing leukocyte recruitment to the site of infection, suggesting that the infection provides intrinsic factors for modulating the host immune response that can interfere with the outcome of sepsis. This phenomenon can be possible because the inflammation developed during *T. gondii* infection is maintained during its chronic phase and interferes with the outcome of the secondary infection caused by CLP. Interestingly, this phenomenon is specific of *T. gondii*, since other protozoa as *Trypanosoma cruzi, Plasmodium chabaudi*, or even a fungus, *Paracoccidiodes brasiliensis*, do not induce an increased susceptibility to sepsis in mice.

It is well known that CD4^+^ and CD8^+^ memory T cells are essential for the control of *T. gondii* proliferation, preventing the de-encystation and reactivation of the disease (Gazzinelli et al., 1992). Our findings revealed that *T. gondii* infection robustly induces long-lived IFN-γ- and TNF-α-producing CD4^+^ and CD8^+^ T cells that are maintained, reprogrammed and amplified to act against a secondary challenge of sepsis. Our data suggests that central memory-like T cells are induced and maintained in the secondary lymphoid organs during chronic *T. gondii* infection by expressing the surface receptors CCR7 and CD62L. After sepsis induction, such cells are reprogrammed and quickly converted to effector memory-like cells by reducing their expression of both CCR7 and CD62L, which facilitates their migration to peripheral organs where they produce robust amounts of NO, IFN-γ and TNF-α to control the bacterial burden.

There are several factors that must be considered when analyzing *T. gondii* oral infection. During its acute phase, intestinal injury is responsible for the transient release of bacterial microbiota in the mesenteric lymph nodes, spleen, and liver, thereby triggering the activation and differentiation of memory cells that are specific not only for the parasite but also for antigens of the microbiota (Benson et al., 2009; Hand et al., 2012). Nevertheless, we showed that bacterial translocation did not influence the outcome of sepsis severity because intestinal damage induced by DSS did not induce memory cells or interfere with the host survival after CLP. Thus, the presence of long-lived *T. gondii*-experienced CD4^+^ and CD8^+^ T cells was more influential in aggravating sepsis than bacterial translocation.

Oral *T. gondii* infection induces highly inflammatory responses that dysregulate the intestinal epithelium and cause ileitis (Craven et al., 2012). Our findings support that inflammation due to chronic *T. gondii* infection aggravated the intestinal tissue damage after CLP. Such characteristic features promote a synergistic cytokine effect that can lead to septic shock (Fong et al., 1989; Calandra and Glauser, 1990; Dofferhoff et al., 1991; Dinarello, 1997). Chronic *T. gondii* infection promoted the overexpression of IFN-γ-related genes, which were exacerbated by CLP. Indeed, the blockade of IFN-γ prevented hypotension and improved the host survival upon sepsis induction.

Recent studies have shown that epigenetic changes, such as methylation, can induce gene silencing and heterochromatin remodeling that inhibits the access of transcription factors to DNA (Jaenisch and Bird, 2003; Esteller, 2007; Gómez-Díaz et al., 2012; Fernández-Sánchez et al., 2013). Here, *T. gondii* infection promoted the methylation of the *gata3* gene, which inhibited access to this gene and impaired the Th2 immune response, what could explain the absence of IL-4 in the peritoneum. IL-10 apparently does not take part of the immunoregulation, since it is produced equally in not infected or infected groups. In contrast, the *Tbx21* and *Eomes* genes were not inhibited by the methylation process, which facilitated the induction of the Th1 immune response and its maintenance during sepsis. Interestingly, naïve C57BL/6 mice exhibited 47% *gata3* methylation, which explains in part why such mice strains have an intrinsic susceptibility to pathogens that promote the Th1 immune response.

Since the overproduction of inflammatory cytokines is the primary cause of mortality in septic patients (Puneet et al., 2010), we reduced the robust inflammatory response using an IFN-γ monoclonal antibody. When subjects were treated with low doses of anti-IFN-γ, we observed a significant improvement in the survival rate of septic mice that were previously infected by *T. gondii*. These results support that although this cytokine is necessary to control chronic *T. gondii* infection (Gazzinelli et al., 1994), the overproduction of it may contribute to sepsis severity.

Robustness of systemic inflammation is the main factor that aggravates sepsis and predisposes patients to septic shock (Tumes et al., 2013). Our findings showed that previous exposure to *T. gondii* was a factor that intensified IFN-γ production and aggravated sepsis severity. Patients previously exposed to *T. gondii* had an increased mortality rate during sepsis compared to non-exposed patients. In this study, we proposed that serology for toxoplasmosis should be monitored in septic patients to predict sepsis severity. Because serology for toxoplasmosis is not time-consuming and laborious, it could be used to optimize the screening of sepsis severity.

In conclusion, we described that toxoplasmosis imprints intracellular signals that activate CD4^+^ and CD8^+^ T cells to produce IFN-γ. During infection, such cells are converted to memory-like T cells to maintain a pool of central memory-like CD4^+^ T cells in secondary lymphoid organs. Our data suggest that during sepsis induction, central memory-like T cells specific to *T. gondii* are properly converted to effector memory-like T cells. Although their specificity against *T. gondii* is not impaired, such cells can cross-react against bacteria to control secondary bacterial infections. Nevertheless, the exacerbated systemic inflammatory response is deleterious to the host because it aggravates SIRS, leading to hypotension with consequent septic shock. Collectively, these data demonstrate that environmental features, such as previous chronic and non-lethal infections, may explain why sepsis has a broad spectrum of clinical forms.

## Author contributions

MCS, DF, AK, LB, JA, FC, RG, and JS designed the study. MCS, DF, AK, LB, GB, MD, WA, and MABS performed the mouse experiments. MCS, AG, and MB provided the samples and performed the patient's experiments. MCS, AK, and TM wrote the manuscript. MCS, LB, AK, RG, and JS edited the manuscript.

## Funding

This work was supported by the Coordination for the Improvement of Higher Education Personnel (CAPES) and the National Counsel of Technological and Scientific Development (CNPq). “The research leading to these results received funding from the Sao Paulo Research Foundation (FAPESP) under grant agreement n 2013/08216-2 (Center for Research in Inflammatory Disease), from the University of Sao Paulo NAP-DIN under grant agreement no. 11.1.21625.01.0.”

### Conflict of interest statement

The authors declare that the research was conducted in the absence of any commercial or financial relationships that could be construed as a potential conflict of interest.
