# Supplementary material for: Chronic Toxoplasma gondii Infection Exacerbates Secondary Polymicrobial Sepsis
Source: Front Cell Infect Microbiol. 2017 Apr 7;7:116. doi: 10.3389/fcimb.2017.00116 (PMC5383667; doi:10.3389/fcimb.2017.00116)
Supplement: Supplementary file 2 [file Presentation1.PDF]

Supplementary Material

Chronic Toxoplasma gondii infection exacerbates secondary polymicrobial sepsis

Maria C. Souza1, Denise M. Fonseca1, Alexandre Kanashiro2, Luciana Benevides1, Tiago S. Medina1, Murilo S. Dias1, Warrison A. Andrade3, Giuliano Bonfá1, Marcondes A. B. Silva2, Aline Gozzi4, Marcos C. Borges4, Ricardo T. Gazzinelli3, José C. F. Alves-Filho2, Fernando Q. Cunha2, João S. Silva1*.

*** Correspondence:**

Corresponding author: João Santana Silva – Department of Biochemistry and Immunology – Ribeirão Preto Medical School – University of São Paulo – Av. Bandeirantes, 390 – 14090-900 – Ribeirão Preto – São Paulo – Brazil – Phone/Fax: (55) 16 3315 3234 – jsdsilva@fmrp.usp.br.

Supplementary Material

1.2 Supplementary Material and Methods

1.2.1 DNA Extraction and Quantification of Tissue Parasite Burden

Fragments of brain tissue were collected 24 hours after CLP surgery and stored in a dry tube at -70°C. Genomic DNA was extracted from approximately 30 mg of the tissue or from 10^7 *T. gondii* tachyzoites using the illustra Tissue & Cells GenomicPrep kit (GE Healthcare, Freiburg, Germany). DNA was quantified in a spectrophotometer (Thermo Scientific NanoDrop 1000, Wilmington, DE), and the parasite burden was assessed by quantitative PCR (qPCR) using SYBR Green (Life Technologies, Carlsbad, CA, USA) from 0.1 µg of each sample. The reaction was performed in a Sequence Detection System ABI 7000 (Life Technologies) with the following parameters: 2 min. at 50°C, 2 min. at 95°C, forty cycles of 15 sec. at 95°C, 30 sec. at 58°C and 30 sec. at 72°C, beyond a dissociation step, with temperature ranging from 60 to 95°C. The amplification of the parasite DNA was performed using primer pairs specific for the conserved *T. gondii* B1 gene (sense: 5′-TTC AAG CAG CGT ATT GTC GA-3′ and antisense: 5′-CAT GAA CGG ATG CAG TTC CT-3′ - MWG Oligo Synthesis Report, Miami, FL) that is found in all known parasite strains. The results were obtained based on a standard curve created with graded concentrations of parasite DNA.

1.2.2 Myeloperoxidase (MPO) Activity

The neutrophil recruitment to the ileum was evaluated using the MPO kinetic-colorimetric assay. The samples were homogenized using a Tissue-Tearor (Biospec®) in ice-cold K_2HPO_4 buffer (400 µL, 50 mM, pH 6.0) containing HTAB (0.5% weight/volume), and the homogenates were centrifuged (16,100 g × 2 min × 4 °C). The supernatants (30 µL) were mixed with K_2HPO_4 buffer (200 µL, 50 mM, pH 6.0) containing *o*-dianisidine dihydrochloride (0.0167%, w/v) and hydrogen peroxide

(0.015%, v/v). The absorbance was determined after 5 min at 450 nm (Multiskan GO Microplate Spectrophotometer, Thermo Scientific, Vantaa, Finland). The results of MPO activity are expressed as the number of neutrophils per mg of tissue by using a standard curve of neutrophils (196–400,000 cells).

1.2.3 Gene Expression

The total RNA was extracted from the ileum of mice using a method of harvesting that utilizes Trizol reagent (Invitrogen). After extraction, the RNA quantity and quality were determined by a spectrophotometer (Thermo Scientific NanoDrop 1000). Complementary DNA (cDNA) was synthesized using 2 µg of RNA and reverse transcriptase SuperScript III (Life Technologies) on a thermocycler PTC-100 (MJ Research, Watertown, NY). The cDNA was amplified in a qPCR using SYBR Green (Life Technologies) and gene-specific primer for Ocludin (F: ATGTCCGGCCGATGCTCTC) and (R: TTTGGCTGCTCTTGGGTCTGTAT) in a real-time PCR thermocycler. Cycle threshold (Ct) data were normalized to the expression of the reference gene (GAPDH) and analysed.

1.2.4 Quantification of RNA Expression Using Nanostring

Total spleen cell lysates from C57BL/6 mice were analysed using the NanoString methodology. Briefly, 10,000 cells were resuspended in 1 µl of Qiagen RLT lysis buffer and hybridized to the target specific code set ON at 65 °C. The code set contained probes against a panel of 50 genes encoding relevant innate immunity proteins (Table S1). After incubation, the samples (three animals per group) were loaded onto the NanoString Prep station for excess reporter removal, binding to the cartridge surface, and probe scanning. After scanning, data were collected and entered into a digital analyser and data normalization was performed against positive and negative control oligonucleotides as well as three housekeeping genes (Hprt1, GAPDH, and Gus1). Data were normalized with sham group mice results and presented as the relative mRNA level. Statistical significance was calculated using Student's t-test and set at $p < 0.01$. The quantification of gene expression was evaluated in the total RNA by the nCounter Analysis System, and data were grouped using the MultiExperimentViewer (MeV v4.6.2). The data were normalized using the sham group as a control, followed by the conversion of the data to log2.

1.2.5 Nitrite Production

Samples from the ileum were collected 24 h after CLP surgery and homogenized in 500 µL of saline. Nitrite concentration was determined by the Griess reaction as an indicator of nitric oxide production. Briefly, 100 µL of the homogenate was incubated with 100 µL of the Griess reagent for 5 min at 25 °C, and nitrite concentration was determined by measuring the optical density at 540 nm (Multiskan GO, Thermo Scientific) in reference to a standard curve of a $NaNO_2$ solution. The results are expressed as µmol of nitrite per mg of tissue.

1.3 Supplementary Figures

Supplementary Figure 1. Polymicrobial sepsis does not induce reactivation of chronic infection with *T. gondii*. C57BL/6 mice were infected with 5 cysts of *T. gondii* and subjected to CLP 40 days after infection. The amount of cysts in the brain (A) and quantification of parasite DNA by real-time PCR (B) were assessed 24 hours after CLP. Data are presented as the means ± SEM for 4 animals in

three different experiments. Statistical analysis was performed using ANOVA followed by Tukey's test.

Supplementary Figure 2. The effect of previous *T. gondii* infection on myeloperoxidase (MPO) activity and tight junction protein expression during sepsis is shown. Neutrophil recruitment to the ileum was assessed using the myeloperoxidase (MPO) assay (A), and the relative mRNA expression of occludin (B) in homogenates of the control groups or T. gondii-infected mice subjected to SL-CLP is shown. Data are presented as the means ± SEM for 4 animals in three different experiments. Statistical analysis was performed using ANOVA followed by Tukey's test; **, P <0,01.

Supplementary Figure 3. CD4+ and CD8+ T cells from T. gondii infection remain activated during the chronic phase of infection. Splenocytes of control or coinfected mice were stained for CD3, CD4, CD8, and CD44 (A and B). Statistical analysis was performed using ANOVA followed by Tukey's test; *, P <0,05; **, P <0,01. For nanostring analysis, the RNA from spleen cells was isolated from control or coinfected mice (C). The quantification of gene expression was evaluated in total RNA using the nCounter Analysis System, and the data were grouped using the MultiExperimentViewer (MeV v4.6.2). The data were normalized using the sham group as a control, followed by the conversion of the data to log2.

Supplementary Figure 4. T. gondii infection enhances nitric oxide production during sublethal polymicrobial sepsis. The production of nitric oxide was indirectly measured using the Griess method in the serum (A) and peritoneal lavage (B) of control or coinfected mice and presented here as µM of nitrite. The bars represent the means ± SEM for 4 animals per group. Similar results were obtained in three independent experiments, and the statistical analysis was performed using ANOVA followed by Tukey's test; ***, P <0,001.
