# Supplementary figures and images for: Chronic Toxoplasma gondii Infection Exacerbates Secondary Polymicrobial Sepsis
Source: Front Cell Infect Microbiol. 2017 Apr 7;7:116. doi: 10.3389/fcimb.2017.00116 (PMC5383667; doi:10.3389/fcimb.2017.00116)

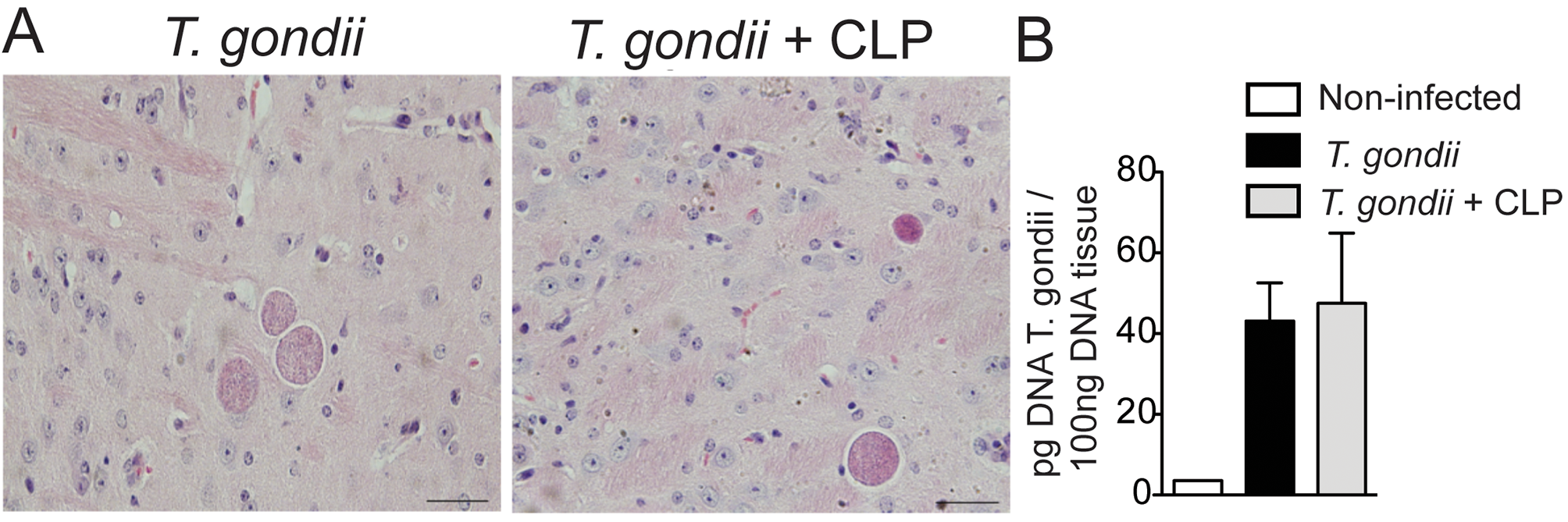

Supplement: Supplementary file 3 [file Image1.TIF]

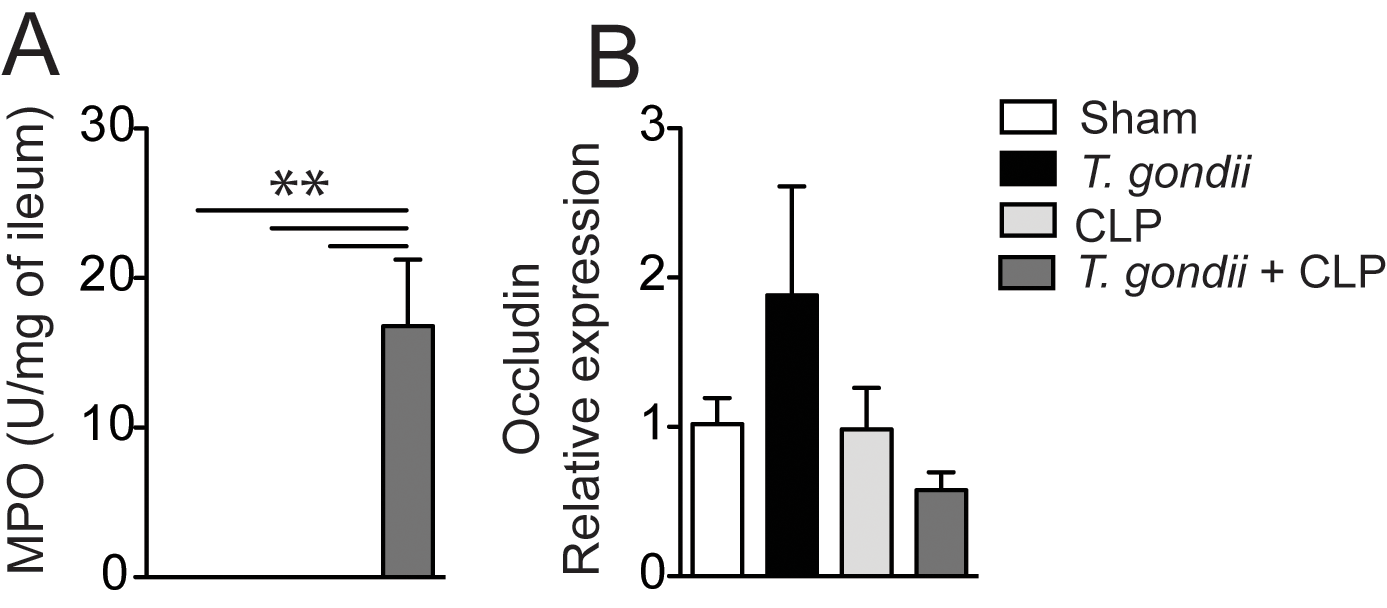

Supplement: Supplementary file 4 [file Image2.TIF]

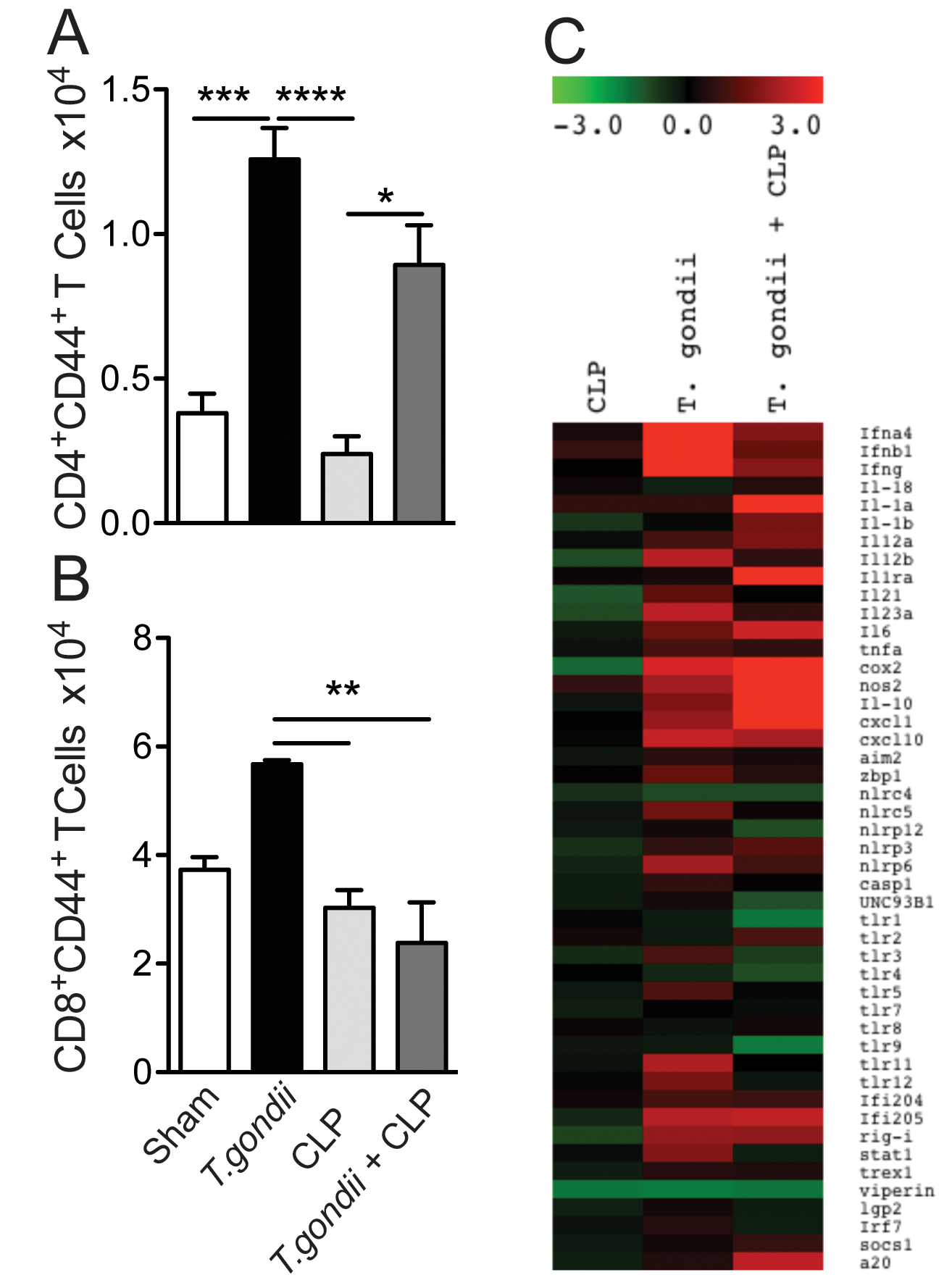

Supplement: Supplementary file 5 [file Image3.TIF]

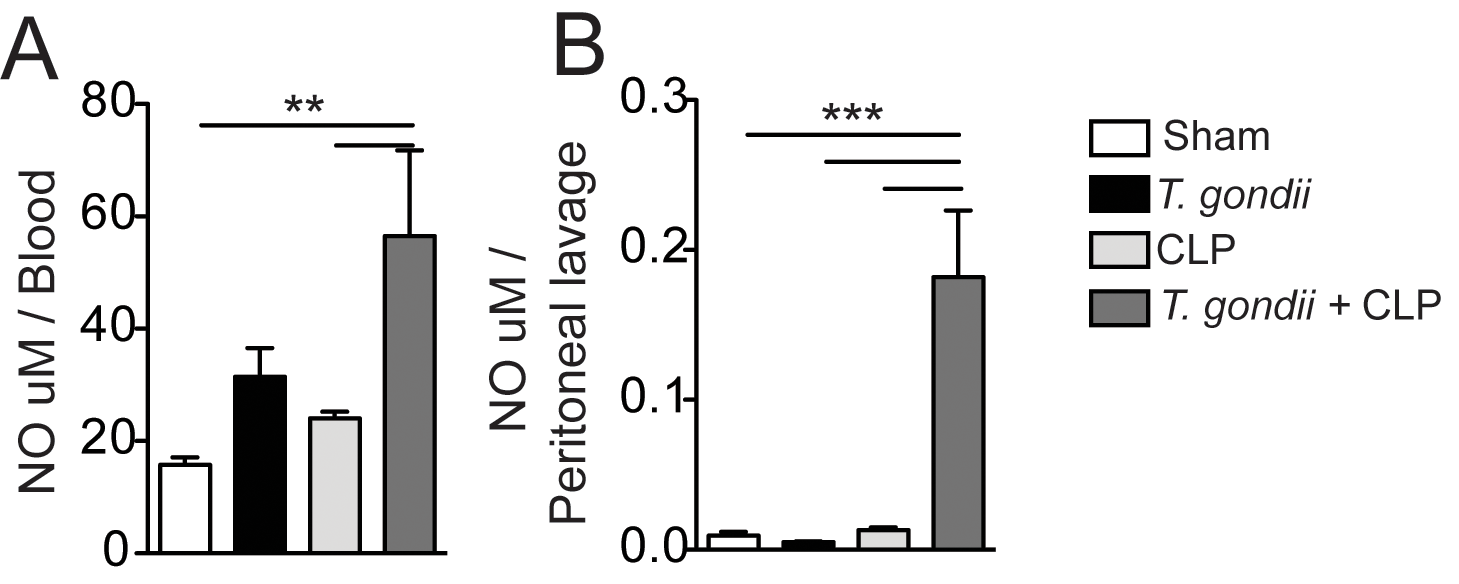

Supplement: Supplementary file 6 [file Image4.TIF]
